# The Effects of Players’ Rotations on High-Intensity Activities in Professional Futsal Players

**DOI:** 10.5114/jhk/169522

**Published:** 2023-10-11

**Authors:** João Nuno Ribeiro, Farzad Yousefian, Jordi Illa, Micael Couceiro, Jaime Sampaio, Bruno Travassos

**Affiliations:** 1 Department of Sport Sciences, University of Beira Interior, Covilhã, Portugal.; 2 Research Centre in Sport Sciences, Health Sciences and Human Development, CIDESD, CreativeLab Research Community, Vila Real, Portugal.; 3 Portugal Football School, Portuguese Football Federation, Oeiras, Portugal.; 4 Sports Performance Area, Sport Science Department, Futbol Club Barcelona, Spain.; 5 Ingeniarius, Lda., Alfena, Portugal.

**Keywords:** physical performance, team sport, substitutions, efforts, rotation duration, rate of fatigue

## Abstract

The current study aimed to investigate the effects of interchange rotations on players` physical performance during competition, with special reference to high-intensity activity (HIA) according to the playing position. Physical performance data, collected from 19 professional players during seven official matches from the Spanish futsal league using a portable local positioning system, included the number of high-speed running activities (>18 km·h^−1^), high-intensity accelerations (>3 m·s^−2^), and high-intensity decelerations (>3 m·s^−2^). Statistically significant (p ≤ 0.05) differences were observed in the number of HIA efforts across rotations and between positions. Players performed more HIA efforts in the first rotation (n = 17.6), suggesting that their first rotation was more demanding than all subsequent rotations. Wingers demonstrated a higher HIA effort and frequency of HIA efforts when compared to defenders (p ≤ 0.05) and pivots (p ≤ 0.001). For all positions, the first rotation was more physically demanding as the number of HIA efforts per rotation decreased with an increased number of rotations throughout the match. Furthermore, higher HIA profile positions, such as wingers and defenders, were less likely to maintain consistent HIA properties (repetition number, time-frequency, and the work-rate) across subsequent rotations during the match. The findings of the study can inform coaching decisions regarding players’ rotations to maintain consistent HIA performance throughout the match.

## Introduction

In intermittent team sports such as futsal (indoor soccer), previous research has shown that to understand players’ performance, it is necessary to consider high demands on players' actions over time as a function of the characteristics of the competition (Ribeiro et al., 2022b). Currently, local positioning systems (LPSs), based on ultra-wideband technology, are utilized to measure external load metrics and physical demands of high-intensity activities (HIAs) during a futsal match (Ribeiro et al., 2020). The collective HIA is composed of the sum of external load variables including mechanical (acceleration and deceleration) and kinematic (speed and distance covered) dimensions, which are measured from high-intensity thresholds. Thus, the combined mechanical and kinematic variables allow for a more holistic analysis of the physical requirements of the game, rather than analyzing the respective performance variables individually. Futsal is an energetically demanding sport, with frequent bouts of repeated sprints, accelerations, and decelerations, interspersed with short recovery periods. Therefore, considering competition demands, the importance of a well-developed aerobic and anaerobic energy system underpins the ability to repeatedly perform HIA throughout a match (Ayarra et al., 2018; Barbero-Alvarez et al., 2008; Krolikowska et al., 2023; Ribeiro et al., 2020; Spyrou et al., 2021; Zerf et al., 2021). Previous literature surrounding football performance has reported a positive association between successful teams and their ability to perform HIA (Di Salvo et al., 2009; Krustrup et al., 2006; Ortega et al., 2022).

In fact, coaches claim that information regarding HIA is required and routinely included in their physical performance reports (Nosek et al., 2021), which inform the development of sport-specific training drills that maximize competition performance (Impellizzeri et al., 2020).

Recent research on the analysis of HIA in futsal competitions has shown that elite futsal players can maintain their physical performance during matches (Illa et al., 2020; Ribeiro et al., 2020; Serrano et al., 2020), between and within matches, and even during congested periods (Ribeiro et al., 2022b). Together with the coaches’ ability to manage player interchange rotations, for which there are no limits in futsal, playing time and rest time (work-to-rest ratio) appears to be one of the most important factors in supporting the players' ability to maintain a high level of performance throughout the game (Dos-Santos et al., 2020; Ribeiro et al., 2022b).

Previous research has shown that teams implementing a high-frequency rotation strategy, with a work-rest ratio close to 1:1 (Barbero-Alvarez et al., 2008; Doğramacı et al., 2015), contributed to maintaining high levels of players’ performance throughout the match (Clay and Clay, 2014; Milanez et al., 2020).

Considering the variability of activity duration and playing time in futsal, not only should the general values of HIA be considered, but also research in other sports that takes into account other HIA characteristics such as the distance covered and the duration of each HIA, the temporal frequency of each action, and the distance covered by players to adjust physiological responses to the training design(Buchheit and Laursen, 2013). Thus, there is a need to identify new metrics for HIA that would consider playing time and rest intervals in each player rotation, which could allow for a more relevant assessment of the overall physical performance according to the various HIA characteristics. To facilitate the transfer of this knowledge to the field, which is essential for the effective design of timed drills, recovery periods, and individualization of a training program, it is also necessary to better understand the responses to HIA in futsal as a function of the playing position and time (Illa et al., 2021; Serrano et al., 2020).

In effect, this approach is expected to provide a comprehensive assessment of position-specific differences in physical performance to inform practitioners working with futsal players to (1) tailor and personalize interventions based on the specific needs of athletes rather than relying on a “one-size-fits-all” approach, (2) better apply position-specific recovery strategies, and (3) strategize player rotations to allow sufficient time between HIA, which function to reduce injury risk and achieve optimal performance outcomes.

Therefore, our research objectives were threefold: 1) to analyze HIA characteristics (the number of efforts, total distance covered, total duration, time-frequency, and the work-rate) by playing position in each player interchange rotation, 2) to investigate the rate of change in the performance work-rate across player interchange rotations for each playing position, and 3) to investigate the effect of interchange rotation duration on various HIA characteristics. We hypothesized that each playing position would have a different profile of HIA characteristics, but with each position expected to maintain their physical performance on the various HIA characteristics throughout the interchange rotations.

## Methods

A retrospective observational study was conducted to quantify and analyze positional differences in high-intensity external load activities of elite futsal players during seven official matches from the premier Spanish Futsal League (2018–2021; n = 266 observations).

### Participants

A convenience sample of data was collected from nineteen professional futsal players (age: 28.8 ± 2.4 years, body mass: 73.7 ± 6.2 kg, body height: 175.9 ± 5.9 cm) who were classified according to their playing position into defenders (DF; *n* = 6), wingers (WG; *n* = 10), and pivots (PV; *n* = 3). Goalkeepers were not included in this study because their position is very specific and their locomotor dynamics are different from the outfield players (Serrano et al., 2021). The monitoring process of players was routine for all players. All participants were informed of the purpose of the study and gave written informed consent before the study was conducted. The experimental procedures used in this study were conducted according to the Declaration of Helsinki and were approved by the Institutional Review Board of the University of Beira Interior (CE-UBI-Pj-2020-043).

### Design and Procedures

Matches were randomly selected while simultaneously controlling various situational factors (phases of the season, all official matches were played on the same indoor court under similar environmental conditions, and team or opponent standards). Matches were included only if they were close games (goal differential ≤2) and were excluded if a player was dismissed, as this can affect overall work-rates (Ju et al., 2022).An LPS performance tracking system (WIMU PRO^TM^, Realtrack Systems, Almeria, Spain) was used to monitor and collect external load data. The LPS devices were placed in the upper part of the back in tight-fitting harnesses. The LPS was installed on the court as follows: 6 antennas with ultra-wideband technology were placed 5 m from the court perimeter line. The intraclass correlation coefficient (ICC)_(2,1)_ 2-way random-effects model was used to analyze the reliability of the absolute agreement of the average measures results. Based on the 95% confident interval of the ICC estimate, reliability was considered acceptable when ICC values were ≥ 0.75 (Koo and Li, 2016).

The local positioning system and ultra-wideband technology had been previously validated with accuracy (bias: 0.57–5.85%), a 1.19 typical error of measurement in test-retest reliability, interunit reliability (bias: 0.18), a high ICC for the x-coordinate (0.65) and a very large ICC for the y-coordinate (0.88), with good 2% of typical error of measurement (Bastida-Castillo et al., 2019), and reported to be reliable for monitoring futsal players (Illa et al., 2021; Ribeiro et al., 2020; Serrano et al., 2020).

The WIMU PRO software (SPRO™, Realtrack Systems, Almeria, Spain) was used to calculate the physical demands of interest in each player rotation.

Players were continuously monitored throughout the match using the LPS, but external load data were only analyzed when players were on the court. It was decided not to analyze the specific data recorded while one of the teams was using a flying goalkeeper (fly Gk + 4 vs. 4 + Gk), since technical-tactical changes may occur during such moments of the match.

### Measures

The duration of each player’s rotation was measured in absolute time (the total time of the match, including when the ball was not in play) to better understand the workload characteristics. Also, the rotation was only considered when players spent more than or equal to 30 s on the field. The following physical demand variables were measured and reported as the number of high-speed running activities (> 18 km•h^−1^), high-intensity accelerations (≥ 3 m•s^−2^), and high-intensity decelerations (≤ −3 m•s^−2^). The sum of these three variables was calculated for each player per rotation to measure the individual HIA per rotation. HIA properties were calculated as (1) the number of HIA efforts (n; HIA effort), (2) the total distance covered in meters for each HIA (m; HIA distance), (3) the duration in seconds of each HIA (s; HIA duration), (4) the time in seconds between each HIA (s; HIA time-frequency), and (5) the total HIA distance covered during the duration of the rotation (m/min; HIA work-rate).

### Statistical Analyses

Normal distribution of data was confirmed using the Shapiro-Wilk test. Differences in mean HIA properties (dependent variables) between rotations, players, as well as any interactions between the independent variables were determined using linear mixed-effects models. Five rotations (R1–R5) and three positions (DF, WG, and PV) were included as fixed effects. A repeated measures model was used to analyze differences between rotations, and players were included as random effects. Bonferroni *post-hoc* tests were applied to identify differences between means in the event of a significant effect.

A mixed effects regression model with random intercepts was used to compare differences in the average rate of change in the HIA number of efforts, HIA frequency, and the HIA work-rate per subsequent interchange rotation between playing positions. HIA performance variables (HIA efforts, HIA frequency, and the HIA work-rate) were included as fixed effects, participants were included as random effects, and the rotation number was treated as a continuous variable. Therefore, the regression slope indicates the average rate of change in the respective HIA performance variable as a function of subsequent interchange rotations. Spearman Rho correlation analysis was used to determine the relationship between HIA properties across rotations. Results of the correlation analysis were interpreted as follows: 0.9–1.0, very high; 0.7–0.9, high, 0.5–0.7, moderate, 0.3–0.5, low; and 0.0–0.3, trivial (Mukaka, 2012). The magnitude of differences between rotations was assessed using Cohen’s *d* effect size, based on the comparison of the difference in means divided by the standardized pooled standard deviation (SD) and adjusted for differences in sample size, and interpreted based on the following criteria: ≥ 0.2–0.5 small, ≥ 0.5–0.08 moderate, and ≥ 0.8 large effect size.Data are presented as estimated marginal mean ± 95% confidence intervals unless otherwise stated. The significance level was set at *p* ≤ 0.05. Statistical analyses were performed using IBM SPSS for Windows statistics, version 25.0 (IBM Corp., Armonk, NY, United States).

## Results

The overall mean duration of each rotation was 5.88 min (5.6–6.2), with WG having the greatest overall rotation time at 6.01 min (5.6–6.4), followed by DF at 5.88 min (5.3–6.5), and 5.52 min (4.7–6.3) for PV.

Table 1 displays HIAs according to players’ positions and interchange rotations. In general, statistically significant (*p* ≤ 0.05) differences were observed in HIA efforts across positions and rotations. WG performed a significantly greater number of HIA efforts overall and across their rotations than both DF (*p* ≤ 0.05; ES = 0.4) and PV (*p* ≤ 0.001; ES = 0.9). Furthermore, WG had a higher total HIA work-rate than PV (*p* ≤ 0.001; ES = 0.8). There were also significant differences in HIA time frequency between positions, with WG (*p* ≤ 0.05; ES = 0.7) and PV (*p* ≤ 0.001; ES = 1.9) demonstrating lower values. Overall, a significantly greater number of HIA efforts was performed during the first rotation (n = 17.64 (16.9–19.4)) compared to subsequent rotations. Similarly, there was a significant effect of the rotation number on the HIA work-rate (*p* ≤ 0.01; ES = 0.6), with the first rotation HIA work-rate being significantly greater than the third (*p* ≤ 0.01; ES = 0.6), the fourth (*p* ≤ 0.05; ES = 0.6) and the fifth rotation (*p* ≤ 0.05; ES = 0.8).

**Table 1:** Estimated Marginal Means [95%CI] in HIA demands for rotations and playing positions.

R Duration (min)		ES	HIA Effort (n)	ES	HIA time-frequency (s)	ES	HIA Duration (s)	ES	HIA Work-Rate (m/min)	ES	HIA Distance (m)	ES
	**R1**	6.73 [6.4 ; 7.1]	-	19.47 [17.9 ; 21.1]	-	21.79 [20.3 ; 23.2]	-	1.73 [1.7 ; 1.8]	-	20.93 [19 ; 22.9]	-	5.36 [5.1 ; 5.6]	-
	**R2**	6.23 [5.7 ; 6.7]	-	16.19 [14.4 ; 18] *	0.5	24.62 [22.5 ; 26.7]	-	1.72 [1.7 ; 1.8]	-	19.26 [16.8 ; 21.7]	-	5.68 [5.4 ; 6]	-
	**R3**	5.48 [4.8 ; 6.2] *	0.6	13.39 [11.5 ; 15.3] **	0.9	24.5 [22.4 ; 26.6]	-	1.77 [1.7 ; 1.8]	-	15.96 [13.6 ; 18.3] **	0,6	5.54 [5.2 ; 5.9]	-
	**R4**	5.3 [4.5 ; 6.1] *	0.7	13.4 [11.6 ; 15.2] **	0.9	23.73 [21.6 ; 25.8]	-	1.76 [1.7 ; 1.8]	-	16.55 [14.1 ; 19] *	0,6	5.97 [5.5 ; 6.4]	-
	**R5**	5.66 [4.7 ; 6.6]	-	13.58 [11.5 ; 15.7] **	0.9	24.3 [21 ; 27.6]	-	1.73 [1.6 ; 1.8]	-	15.32 [12.1 ; 18.5] *	0,8	5.68 [5.1 ; 6.2]	-
	**Total**	5.88 [5.6 ; 6.2]	-	15.2 [14 ; 16.4]	-	23.79 [22.4 ; 25.2]	-	1.74 [1.7 ; 1.8]	-	17.61 [16.1 ; 19.1]	-	5.64 [5.4 ; 5.9]	-
**DF**	**R1**	6.26 [5.6 ; 6.9]	-	17.41 [14.6 ; 20.2] #	2.2	22.21 [20.1 ; 24.3]	-	1.72 [1.6 ; 1.8]	-	19.31 [15.9 ; 22.7]	-	5.27 [4.8 ; 5.7]	-
	**R2**	6.83 [5.9 ; 7.8]	-	17.65 [14.3 ; 21] #	1.7	26.74 [23.5 ; 30]	-	1.68 [1.6 ; 1.8]	-	21.66 [17.3 ; 26.1]	-	5.49 [4.8 ; 6.1]	-
	**R3**	5.91 [4.5 ; 7.3]	-	14.07 [10.6 ; 17.6]	-	27.07 [23.5 ; 30.6]	-	1.71 [1.6 ; 1.8]	-	15.31 [10.9 ; 19.8]	-	5.21 [4.6 ; 5.8]	-
	**R4**	5.6 [4.2 ; 7]	-	12.26 [9 ; 15.5]	-	24.67 [20.6 ; 28.8]	-	1.74 [1.6 ; 1.9]	-	15.3 [11.1 ; 19.5]	-	5.41 [4.5 ; 6.3]	-
	**R5**	4.78 [3.1 ; 6.5]	-	9.42 [6.4 ; 12.5]	-	24.58 [17.9 ; 31.2]	-	1.6 [1.4 ; 1.8]	-	9.58 [4.4 ; 14.7]	-	5.08 [4.1 ; 6]	-
	**Total**	5.88 [5.3 ; 6.5]	-	14.16 [12.3 ; 16]	-	25.05 [22.9 ; 27.2] α	0.9	1.69 [1.6 ; 1.8]	-	16.23 [13.8 ; 18.7]	-	5.29 [4.8 ; 5.7]	-
**WG**	**R1**	6.99 [6.5 ; 7.4]	-	21.85 [19.9 ; 23.8] α β	1.3; 0.7	19.28 [17.8 ; 20.7]	-	1.74 [1.7 ; 1.8]	-	23.39 [20.9 ; 25.9]	-	5.16 [4.5 ; 5.8]	-
	**R2**	5.93 [5.3 ; 6.6]	-	16.73 [14.5 ; 19] **	0.8	22.15 [19.8 ; 24.5]	-	1.72 [1.7 ; 1.8]	-	19.92 [16.9 ; 22.9]	-	6.08 [5.3 ; 6.9]	-
	**R3**	5.03 [4.1 ; 6]	-	13.8 [11.3 ; 16.3] **	1.1	20.87 [18.4 ; 23.4]	-	1.77 [1.7 ; 1.9]	-	16.99 [13.9 ; 20.1]	-	5.81 [4.9 ; 6.7]	-
	**R4**	5.67 [4.6 ; 6,7]	-	15.16 [12.7 ; 17.7] **	1.1	22.11 [19.4 ; 24.8]	-	1.8 [1.7 ; 1.9]	-	18.06 [14.7 ; 21.4]	-	5.67 [4.6 ; 6.8]	-
	**R5**	6.41 [5.2 ; 7.7]	-	17.24 [14.9 ; 19.6] * Δ Φ	0.8; 1.8; 1.8	21.67 [17.1 ; 26.2]	-	1.79 [1.7 ; 1.9]	-	20.05 [16.1 ; 24]	-	4.42 [3.1 ; 5.8]	-
	**Total**	6.01 [.,6 ; 6.4]	-	16.96 [15.7 ; 18.2] α β	0.9; 0.4	21.21 [19.7 ; 22.7] α β	1.9; 0.7	1.77 [1.7 ; 1.8]	-	19.68 [17.9 ; 21.4] α	0.8	5.86 [5.6 ; 6.2]	-
**PV**	**R1**	6.51 [5.6 ; 7.4]	-	13.67 [9.8 ; 17.5]	-	31.16 [28.2 ; 34.1]	-	1.71 [1.6 ; 1.8]	-	14.47 [9.8 ; 19.2]	-	5.46 [5.1 ; 5.8]	-
	**R2**	6.41 [5.1 ; 7.7]	-	11.22 [6.7 ; 15.8]	-	29.3 [23,7 ; 34.9]	-	1.79 [1.7 ; 1.9]	-	11.33 [4.9 ; 17.7]	-	5.66 [5.2 ; 6.1]	-
	**R3**	6.38 [4.6 ; 8.2]	-	10.33 [5.4 ; 15.3]	-	33.31 [27.9 ; 38.7]	-	1.84 [1.7 ; 2]	-	12.65 [6.4 ; 18.9]	-	5.62 [5.2 ; 6.1]	-
	**R4**	3.57 [1.7 ; 5.5]	-	8.99 [4.6 ; 13.3]	-	29.31 [24 ; 34.6]	-	1.69 [1.5 ; 1.9]	-	13.48 [7 ; 20]	-	6.32 [5.7 ; 6.9]	-
	**R5**	4.74 [2.3 ; 7.1]	-	8.52 [4.2 ; 12.9]	-	32.78 [24.6 ; 41]	-	1.76 [1.5 ; 2]	-	10.24 [3 ; 17.5]	-	6.26 [5.6 ; 6.9]	-
	**Total**	5.52 [4.7 ; 6.3]	-	10.55 [8 ; 13.1]	-	31.17 [28.2 ; 34.1]	-	1.76 [1.7 ; 1.8]	-	12.44 [8.9 ; 159]	-	5.43 [4.8 ; 6]	-

R: Rotation, R1: Rotation 1, R2: Rotation 2, R3: Rotation 3, R4: Rotation 4, R5: Rotation 5; DF: Defender, WG: Winger, PV: Pivot; * significantly different than R1 (*p* ≤ 0.05); ** significantly different than R1 (*p* ≤ 0.01); # significantly different than R5 (*p* ≤ 0.01); Δ significantly different than PV (*p* ≤ 0.01); α significantly different than PV (*p* ≤ 0.001); β significantly different than DF (*p* ≤ 0.05); Φ significantly different than DF (*p* ≤ 0.001). ES: Cohen's *d* effect size, reported only for significant differences

The rate of change in HIA variables was considered across five interchange rotations, as it represented the maximum number of rotations observed in the data set. The estimates of fixed effects, which represented the mean initial rotation, for the number of HIA efforts (n) was significantly (*p* ≤ 0.001) lower for PV when compared to DF and WG (PV: 13.2 ± 3.6; DF: 18.26 ± 2.6; and WG: 19.9 ± 1.8). Mean initial rotation HIA frequency was significantly lower for WG (19.63 ± 1.4 s) and the highest for PV (*p* ≤ 0.001; 30.96 ± 2.8 s) when compared to DF (*p* ≤ 0.05; 23.04 ± 2 s). The mean initial rotation HIA work-rate (m/min) was significantly lower (*p* ≤ 0.001) for PV when compared to DF and WG (PV: 13.92 ± 4.2 m/min; DF: 20.76 ± 3 m/min; WG: 22.09 ± 2.1 m/min).

**Figure 1:**
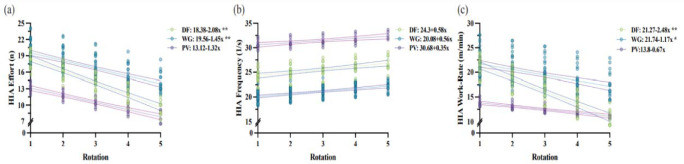
Linear mixed effects regression model equations and predicted values for the rate of change in (a) HIA efforts (n), (b) HIA frequency (1/s), and (c) the HIA work-rate (WR; m/min) between playing positions as a function of the interchange rotation number.

No significant (*p* ≥ 0.05) differences were observed in the slopes of HIA properties between playing positions. However, HIA efforts and the HIA work-rate revealed changes in the slope across subsequent interchange rotations for DF and WG, while a significant increase in the slope was observed for HIA frequency for DF and WG (Figure 1).

The relationship between rotation duration and HIA properties is presented in Table 2. Rotation duration revealed a significant positive moderate correlation between HIA efforts and the HIA work-rate. Furthermore, the HIA work-rate revealed statistically significant positive low and moderate correlations with all of the variables with exception of HIA time-frequency, which revealed a low negative significant correlation.

**Table 2:** Spearman Rho Correlation of HIA properties.

	R Duration (min)	HIA Effort (n)	HIA Time-Frequency (s)	HIA Duration (s)	HIA WR (m/min)
HIA Effort (n)	0.732**	-			
HIA Time-Frequency (s)	0.164*	−0.457**	-		
HIA Duration (s)	−0.041	0.002	−0.087	-	
HIA WR (m/min)	0.598**	0.896**	−0.454**	0.347**	-
HIA Distance (m)	−0.051	0.08	−0.159*	0.852**	0.478**

Significant correlation * *p* ≤ 0.05; ** *p* ≤ 0.01

## Discussion

The purposes of our study were to (1) analyze the HIA properties (the number of efforts, total distance covered, total duration, time-frequency, and the work-rate) of player interchange rotations according to the playing position, (2) to investigate the rate of change in the performance work-rate across player interchange rotations for each playing position, and (3) to investigate the influence of rotation duration on different HIA properties. In general, the results revealed variations in some HIA properties across positions and rotations. Interestingly, HIAs did not reveal changes in the rates of change (slope) between positions, but only across interchange rotations within positions including DF and WG.

In line with our initial hypotheses, rotation duration revealed a significant relationship with HIA properties, namely HIA efforts and the HIA work-rate.

### HIAs Are Dependent on Playing Positions

The primary findings of this study reveal that based on the analysis of HIAs (the number of efforts, time-frequency, and the work-rate), physical demands during elite futsal competition are position-dependent. HIA duration and distance covered did not reveal any differences according to playing positions. These findings are consistent with previous research, in which the authors reported similar values of physical demands between positions based on mean values (Serrano et al., 2020), as well as the most demanding scenarios (Illa et al., 2021). In fact, it may be assumed that HIA duration and distance covered could be defined as variables that characterize general match demands, while HIA efforts, the work-rate and time-frequency as variables that define the structure of match demands according to playing positions.

The interpretation of these findings strengthens the evidence that a futsal player requires a high capacity to perform repeated HIAs, implying that the activity profile of elite futsal players is dependent on both the aerobic and anaerobic energy metabolism pathways, particularly the phosphagen system (Castagna and Álvarez, 2010). Behind this scope, our results are consistent with the literature on energy systems, which highlights the importance of developing a training program that specifically emphasizes the HIA work-rate (energy system), to play elite futsal. During passive recovery, 50 percent of muscle adenosine triphosphate-phosphocreatine (ATP-CP) stores are restored within 20 s of rest and intramuscular reserves are restored after almost 3 min (Bogdanis et al., 1996; Ulupınar et al., 2021). Thus, the ATP-CP replenishing time is similar to the average duration between HIAs, and the restoration of intramuscular reserves is approximately within the average time that elite futsal players are on the bench (Ribeiro et al., 2022a). It is therefore plausible to suggest that futsal players can maintain consistency in performing HIAs throughout a match and during their rotation (playing time) due to the intermittent nature of the sport as well as rest intervals afforded between rotations. However, some caution is required when interpreting this association, because the time between HIAs is not spent passively resting, but rather engaging in less intense activities.

In line with this, despite the similarities between the duration and distance covered per HIA effort, match demands of futsal players differ according to playing positions (Ribeiro et al., 2020). The WG, as expected, was the most demanding position, with higher values of HIAs followed by DF and PV positions. These findings could be explained by the fact that WG usually play with constant variations in the space of play to explore 1 vs. 1 situations or to create numerical and positional superiorities and promote defensive imbalances (Ohmuro et al., 2020; Santos et al., 2020). This evidence is consistent with the findings of previous research, which investigated the areas of the field covered by players and concluded that WG occupied more court space areas in the middle pitch line and with more variability than other playing positions (Serrano et al., 2021).

### Work-Rate Performance Changes across Player Rotations

One of the most difficult challenges in indoor team sports is detecting fatigue patterns. Unlimited substitutions in the match influence fatigue management during the competition. One of the possible outcomes is that elite futsal players have a work-rest ratio (playing time-to-rest time) near 1:1 (Barbero-Alvarez et al., 2008; Doğramacı et al., 2015; Ribeiro et al., 2022b).

With this finding in mind, we observed the effects of subsequent interchange rotations to confirm the loss or gain variation in the different HIAs. The results were very interesting, revealing significant losses (all positions except the PV) in all HIAs (effort, time frequency, and the work-rate) per subsequent rotation compared to the initial rotation.

This phenomenon can occur because the game pace may decrease in the players’ final rotation, towards the end of the match, as a result of tactical strategies (e.g., advantageous/disadvantageous score line, match score, or accrued match fouls). Furthermore, temporary declines in physical performance could be related to the increased amount of time the ball is out of play and thus fewer opportunities to engage in match activities (Carling and Dupont, 2011). Moreover, fatigue is not caused by a single factor, and the sustained decline in HIA performance of WG and DF (higher profile) compared to the first rotation could be associated with a decrease in muscle glycogen (Krustrup et al., 2006) or an increase in potassium in the muscle (Mohr et al., 2005). In soccer, research has found that players with higher physical demands in the first half lower their physical performance in the second half when compared to players with low and moderate physical profiles in the first half (Bradley and Noakes, 2013). This evidence is especially significant because different positions have different activity profiles and probably different bioenergetic requirements.

It is acknowledged that HIA may be influenced by other contextual factors (Novak et al., 2021), and in consequence, we cannot conclude that any decrease in physical performance observed is due to the development of fatigue. However, in futsal, the final result is usually determined in the last moments of the game, resulting in a period marked by high physical and emotional demands, as well as different variations in game dynamics (Méndez-Domínguez et al., 2019). These findings highlight the importance of further studies investigating several contexts to gain a better understanding of HIAs in elite futsal.

### Effects of Rotation Duration on HIA Properties

Playing time is considered a performance factor, especially in sports where substitutions are unlimited and *ball-in-play* time is considered important (Ribeiro et al., 2022b). In this regard, and in response to a growing interest in match demands and their impact on match outcomes, practitioners have begun to monitor the duration of players’ interchange rotations (Ribeiro et al., 2022a).

Interestingly, the structural properties of match-play demands (effort, time-frequency, and the work-rate) revealed strong correlations with rotation duration. This evidence supports previous findings and emphasizes the significance of the relationship between playing time and rest time (work-to-rest ratio) (Ribeiro et al., 2022a). Furthermore, the strong negative association between HIA effort and time-frequency, as well as the strong positive correlation between HIA effort and the work-rate, support this statement. To be more specific, as players increase their HIA effort in match-play, the time between HIA decreases, increasing their workload intensity and, as a result, increasing the distance covered per minute during the duration of the rotation. This evidence is supported by results observed for WG, concerning the overall match and the first rotation (the most demanding), when compared to other positions. While acknowledging differences in modalities, this trend was consistent with other research findings, which concluded that HIAs by professional soccer players occurred within the first 15 min of the match (Oliva-Lozano et al., 2021).

## Practical Implications

The combination of our findings provides critical insight into training prescription, especially when considering positional requirements and adapting these activities to prepare players for such high-demanding competition exposures (Illa et al., 2021). In particular, the findings of this study can contribute to informing the creation of simulation matches or drills based on the specific HIA performance characteristics of a player`s position and rotation during a futsal match.

**Figure 2:**
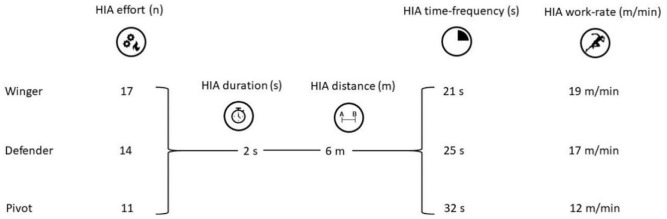
HIA mean values per interchange rotation in different playing positions.

These repeated bouts (Figure 2) should be incorporated into drills for approximately 6-min periods, which represent the average absolute playing time limits per rotation and should be used for the development of more ecologically valid futsal physical tests that incorporate HIAs interspersed with different work-rest ratios.

In addition, analyzing the rate of change in HIA performance could be used as a performance indicator to monitor playing positions or even changes in individual performance across the season. It is expected that such information can support decision-making processes for the prescription and manipulation of training loads during training sessions as well as in competition.

## Conclusions

Our findings suggest that physical demands in futsal are position and time-dependent. Coaches should optimize training sessions to prepare their team to train together not only according to the overall values of the match demands, but also according to the physical demands of each position to individualize their specific needs. Such differences are likely to affect performance and injury risk.

Interestingly, HIA duration and distance are relevant properties to characterize activities with high intensity, while effort, time-frequency and the work-rate are relevant properties to characterize the structure of match-play demands. Moreover, our findings suggest combining the HIA efforts with the HIA work-rate to monitor the training load of a player, a group of players during a given rotation, and the whole team.

According to player rotation analyses, the first rotation is more physically demanding in all positions. A decrease in the ability to perform HIAs (effort, time-frequency, and the work-rate) was observed with an increase in the number of rotations, for the higher activity profile positions (WG and DF), across all rates of performance change analyses. Even though this evidence provides novel insights into the physical demands of team sports with unlimited substitutions, some caution is required when interpreting these findings due to the limitations of the current study. To begin, despite using data from three different seasons, the study sample was limited to one professional futsal team. Furthermore, the fact that there were only three players for the PV position makes the generalization of position results difficult. Lastly, to the best of our knowledge, this is the first study to present information on different HIAs per playing position and player rotation in futsal, which makes it difficult to compare with the literature.
